# Increased Conidia Production and Germination In Vitro Correlate with Virulence Enhancement in *Fusarium oxysporum* f. sp. *cucumerinum*

**DOI:** 10.3390/jof9080847

**Published:** 2023-08-14

**Authors:** Md. Jamal Uddin, Xiaoqing Huang, Xiaohong Lu, Shidong Li

**Affiliations:** 1 Institute of Plant Protection, Chinese Academy of Agricultural Sciences, Beijing 100193, China; jamal_ag@yahoo.com (M.J.U.); huangxiaoqing@caas.cn (X.H.); 2 Crops Division, Bangladesh Agricultural Research Council (BARC), Dhaka 1215, Bangladesh

**Keywords:** *Fusarium oxysporum*, virulence evolution, conidiation, Fusarium wilt, colonization

## Abstract

Cucumber plants commonly suffer from Fusarium wilt disease, which is caused by *Fusarium oxysporum* f. sp. *cucumerinum* (*Foc*). Although resistant cultivars assist with Fusarium wilt disease control, enhancement of the virulence of *Foc* has been identified after monoculture of wilt-resistant cultivars. To investigate the biological characteristics that contribute to the virulence evolution of *Foc*, a wildtype strain foc-3b (WT) and its virulence-enhanced variant Ra-4 (InVir) were compared in terms of their growth, reproduction, stress tolerance, and colonization in cucumber plants. The InVir strain showed similar culture characteristics on PDA media to the WT strain but produced significantly more conidia (>two fold), with a distinctly higher germination rate (>four fold) than the WT strain. The colony diameter of the InVir strain increased faster than the WT strain on PDA plates; however, the mycelia dry weight of the InVir was significantly lower (<70%) than that of the WT harvested from PDB. The InVir strain exhibited a significant increase in tolerance to osmolality (1 M NaCl, 1 M KCl, etc.). The GFP-labeled InVir strain propagated in the cucumber vascular faster than the WT strain. These results suggest that increased conidia production and germination in vitro may correlate with virulence enhancement in *Fusarium oxysporum* f. sp. *cucumerinum*. This study will provide an insight into its virulence evolution and help us understand the mechanisms underlying the evolutionary biology of *F. oxysporum*.

## 1. Introduction

The *Fusarium oxysporum* Schlechtend. species complex is composed of pathogenic and nonpathogenic fungi [1]. Generally, *F. oxysporum* is nonpathogenic in native plant communities, though agricultural practices have provided lots of opportunities for it to become aggressive [2]. As a plant pathogen, *F. oxysporum* can infect more than 100 hosts, and commonly undergoes worldwide distribution [3]. It invades roots and can cause wilt disease symptoms or crown, stem, and root rot. Cucumber (*Cucumis sativus*) commonly suffers from Fusarium wilt disease caused by *F. oxysporum* f. sp. *cucumerinum* Owen (*Foc*) [4]. The incidence of cucumber Fusarium wilt ranges from 10% to 30% yearly for over 1.2 million ha of cucumber planted in China, causing significant yield and economic losses [5,6]. As a typical soilborne disease, cucumber monoculture could increase Fusarium wilt disease incidence up to 70%, and result in a 10 to 50% yield loss [7]. 

No sexual structures have been found under natural or controlled conditions in *F. oxysporum*. All spores produced by *F. oxysporum* include microconidia, macroconidia, and chlamydospores. These asexual spores play important roles during infection. Like most fungi, *F. oxysporum* produces abundant conidia responsible for dispersal and survival [8]. Sporulation in the xylem allows rapid upward movement with the transpiration stream [2]. Furthermore, the pathogenesis process has been proven to be spore density dependent in filamentous fungi [9,10]. Apparently, sporulation capacity is a key factor for a fungal plant pathogen. Chlamydospores are important contributors to the long-time persistence of *F. oxysporum*. 

Currently, there are a lack of effective curative measures for Fusarium wilt disease control. Planting or grafting resistant cultivars or rootstocks are the only practical and environmentally friendly strategies for controlling cucumber Fusarium wilt disease [11]. In fact, monoculture systems in commercial cucumber greenhouses increased soilborne plant diseases, especially Fusarium wilt disease caused by *Foc*, and decreased the control efficacy that benefited from the resistant cultivar. Besides the accumulation of pathogens in the soil, the enhancement of virulence of *Foc* has been proven after serial passage on resistant cultivars [12]. Virulence enhancement has been identified in other formae specials, including *ciceris*, *conglutinans*, and *vasinfectum*, causing chickpea, cabbage, and cotton Fusarium wilt diseases, respectively [13,14,15]. Agricultural practices provide many opportunities for *F. oxysporum* to become more abundant and widespread [2]. 

Apparently, the evolution of virulence enhancement as an adaptive trait is driven by the selective pressure imposed by hosts. How pathogens evolve novel virulence activities has been voted one of the top 10 unanswered questions [16,17]. To figure out how the pathogen *Foc* evolved with increased virulence, we have identified some pathogenic genes and transposases likely to be involved in aggressiveness via transcriptome analysis of virulence-differentiated *Foc* isolates [18]. However, it is hard to directly connect these pathogenic genes with virulence, since the function of most genes is still unknown, whereas it has been found that some biological characteristics, such as mycelial growth, might facilitate the virulence in some fungal pathogens [19]. Conversely, the reduced virulence in *Aspergillus fumigatus* is linked to a decrease in growth rate [20]. Moreover, *Zymoseptoria tritici* strains with ample conidia production usually predominate in mixed infection [21]. However, the specific biological characteristics in the evolution of virulence in *Foc* remain unclear.

To investigate the biological characteristics which contribute to the virulence enhancement of *Foc* under the selective pressure of Fusarium wilt-resistant cultivars, a wildtype strain foc-3b (WT) and its virulence-enhanced variant Ra-4 (InVir) were compared in terms of their growth, reproduction, stress tolerance, virulence, and infection processes. This study will provide an insight into the connection between virulence evolution and biological fitness and help readers understand the mechanisms underlying the evolutionary biology of *F. oxysporum*. 

## 2. Materials and Methods

### 2.1. Strains

The wildtype strain foc-3b (WT, strain number: ACCC39326) with mild virulence and its virulence-enhanced variant Ra-4 (InVir) derived by four serial passages through a resistant cucumber cultivar were obtained in our previous study [12]. GFP-labeled strains of foc-3 (WT) and Ra-4 (InVir) were obtained according to the method described previously [18], and verified by PCR and DNA sequencing, followed by observation under a fluorescence microscope (ZEISS Confocal LSM980, Carl Zeiss Microscopy GmbH, Jena, Germany) with excitation at 488 nm, and detection of emissions at 498–547 nm. Furthermore, GFP-labeled strains had no significant differences in virulence with their respective parental strain. All strains were stored at −80 °C in 30% glycerin.

### 2.2. Morphological Observation

Fungal strains were grown on potato dextrose agar media (PDA) at 26 °C in the dark for seven days prior to observation. Culture characteristics, including texture, density, color, growth front, and zonation, were visually examined. Microscopic observation of the morphology of fungal strains was also conducted under a microscope (BX41, Olympus, Tokyo, Japan). Typical structures, including hyphae, microconidia, macroconidia, and chlamydospore, were observed for each strain.

### 2.3. Mycelia Growth

Two methods were used to determine mycelia growth. In the first method, fungal strains were cultured on PDA in Petri dishes at 26 °C or 28 °C in the dark. Each dish was inoculated with a mycelial plug (4 mm in diameter) taken from the edge of actively growing colonies with a cork borer; there were four dishes per strain. Colony diameters were measured 3–9 days post inoculation. In the second method, fungal strains were cultured in potato dextrose broth (PDB) at 26 °C on a shaker with 180 runs/min. Mycelia were harvested 5 days post inoculation via filtering with 3 layers of sterilized miracloths to remove conidia and broth, washed 3 times using sterilized distilled water (SDW) to remove the residual media, put on Petri dishes, and then dried in a hot air oven at 70 °C for 12 h. The dry mycelia weight of each strain was calculated and expressed as mg/mL. Each experiment was repeated once.

### 2.4. Conidia Production and Germination

Conidia production was measured after being cultured with shaking of 180 runs/min at 26 °C or 28 °C, for 3 to 9 days in PDB and Armstrong medium without agar, respectively. The concentration of conidia was determined using a hemacytometer. Conidia germination was measured in sterile distilled water and on PDA medium. First, the conidia suspension was adjusted to 1.0 × 10^6^ conidia/mL. Then, 20 μL of conidia suspension was placed on a sterilized glass slide with or without PDA medium (area in 1 cm × 2 cm) and covered with plastic cover slips. Following 12 h of incubation at 26 °C in the dark, the conidial germination was examined under a microscope. For each strain, at least 100 conidia were examined per replicate. Each experiment was repeated once.

### 2.5. Stress Tolerance

To determine the stress response, strains were cultured at 26 °C in the dark on PDA plates amended with different stress agents (1 M NaCl, 1 M KCl, 1 M glycerin, 1 M sorbitol, 0.03% sodium dodecyl sulphate (SDS), 30 mM H_2_O_2_, 0.05% congo red (CR), 0.3 mg/L calcofluor white (CFW), 0.2 M CaCl_2_). PDA plates without any other agents were conducted as control plates. Six plates per stress agent were conducted for each strain. The colony diameter of each strain was measured post-7 days incubation. The experiment was repeated once.

### 2.6. Virulence

Cucumber cultivars Zhongnong No. 6 (ZN6, susceptible to *Foc*) and Zhongnong No. 106 (ZN106, moderately resistant to *Foc*) were provided by the Institute of Vegetables and Flowers, CAAS. *Foc* inoculum was created as described above, and conidia suspension with a concentration of 1.0 × 10^6^ conidia/mL was prepared. Healthy seeds of both cultivars were surface sterilized in 2% sodium hypochlorite (NaClO) for 5 min, followed by washing 3 times with sterilized distilled water (SDW), and then kept in a 9 cm Petri dish covered with sterile wet filter paper at 28 °C in the dark for 24 h. Then, two methods, seed soaking and root dipping, were used for inoculation. During seed soaking, 50 pre-germinated seeds were soaked in the conidia suspension for 30 min for each strain; being soaked in SDW was used as a control treatment. The inoculated seeds were sown in the individual holes of a seed tray filled with sterilized substrate (a mixture of vermiculite, peat, and pearlite (1:1:1, *v*/*v*/*v*), autoclaved twice at 121 °C for one hour in a two-day period), and grown in a greenhouse maintaining a 16 h photoperiod at 28/20 ± 1 °C day/night, respectively. The experiment was repeated three times. The disease incidence and disease index of the Fusarium wilt was investigated 14 days post-inoculation (dpi) using a 0 (no symptoms) to 5 (dead seedlings) grade scale, as previously reported [12,22]. For root dipping, pre-germinated seeds were sown in sterilized substrate and incubated in the same conditions as in the seed soaking method. After 14 days incubation, seedlings were dug up and carefully removed from the seed trays. The seedling roots were washed with running tap water followed by SDW, and then surface sterilized with 1% NaClO for 2–3 min followed by rinsing 4–5 times with SDW. Then, the roots were placed on sterilized filter papers so the water could be absorbed, dipped in the conidia suspension of each strain separately for 15 min, and then re-planted in individual plastic pots filled with sterilized substrate, respectively. Seedling roots dipped in SDW served as controls. A minimum of 12 seedlings were used for each treatment. The disease incidence and disease index were assessed four weeks after inoculation. The whole experiment was repeated three times. The disease incidence (DI) and disease severity index (DSI) were calculated as follows: DI = (Number of infected plants/Total number of inspected plants) × 100%; DSI = [Σ (Class × Number of plants in that class)/(Highest disease grade × Total number of inspected plants)] × 100.

### 2.7. Colonization in Cucumber

Two GFP-labeled strains, gWT and gInVir, were used to infect cucumber seedlings for colonization observation. The root-dipping method was used for inoculation, as described above. Seven days post inoculation, three roots were uprooted carefully from the pots for each cultivar and strain pair and washed with SDW to remove adhering surface particles. Tap roots (apex/tip portion, 4–5 mm in length approximately, whole part without sectioning), hypocotyl (between the root and the cotyledon), and epicotyl (between the cotyledon and the first true leaf) were microscopically examined as described above. The hypocotyl and epicotyl were sectioned transversely by hand using disinfected razor blades with very thin layers (20–30 µm approximately).

### 2.8. Statistical Analysis

All data analyses were conducted using SPSS 20 (SPSS Inc., Chicago, IL, USA). One-way ANOVA was used to perform an analysis of variance. A *t*-test was performed to compare the means between the two repeated trials and evaluate the differences in biological characteristics between the WT and InVir strains.

## 3. Results

### 3.1. The InVir Strain Showed Slight Morphological Changes

**Figure 1:**
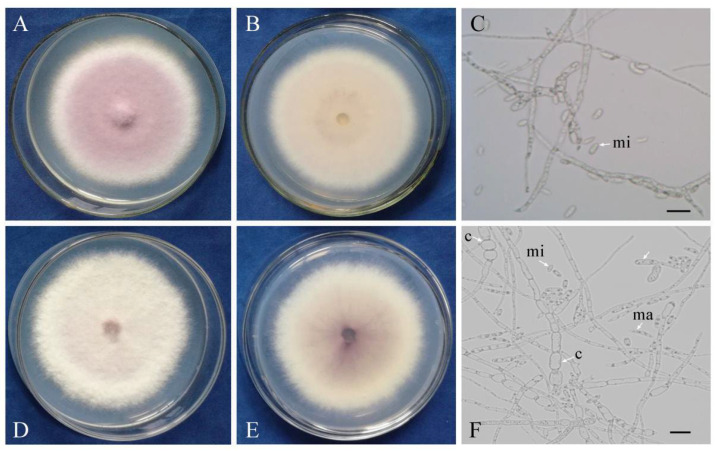
Morphological characters of the wildtype strain foc-3b (**A**–**C**) with mild virulence and its virulence-enhanced variant Ra-4 (**D**–**F**) grown on PDA medium at 26 °C. Front (**A**,**D**) and reverse (**B**,**E**) sides of colonies. mi: microconidia; ma: macroconidia; c: chlamydospore. Scale bar = 20 µm.

The WT and the InVir strains showed similar culture characteristics, but with differences in pigment accumulation (Figure 1). Both WT and InVir had felty mycelium with average density. The surface on PDA of the WT strain foc-3b appeared as a grayish rose, while its reverse side displayed a grayish white. On the other hand, the PDA surface of the InVir strain Ra-4 exhibited a grayish white appearance, while the reverse side appeared reddish gray. Typical structures of microconidia, macroconidia, and chlamydospores were observed in both strains. Notably, the WT strain usually produced more microconidia than macroconidia; in contrast, the InVir produced more macroconidia than microconidia. Chlamydospores were more prevalent in the InVir strain compared to the WT strain.

### 3.2. Mycelia of the InVir Strain Grew Faster Than the WT Strain

**Figure 2:**
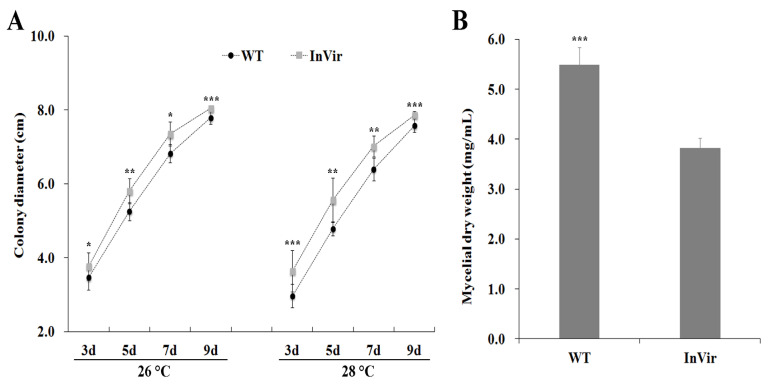
Mycelia growth of the wildtype strain foc-3b with mild virulence (WT) and its variant Ra-4 with increased virulence (InVir). (**A**) Diameters of colonies grown on PDA medium at 26 °C and 28 °C, respectively. (**B**) Dry weight mycelia harvested from PDB medium cultured at 26 °C on a shaker with 180 runs/min for five days. Significant differences indicated by “*” (*p* < 0.05), “**” (*p* < 0.01), and “***” (*p* < 0.0001).

Mycelia growth of the InVir strain was faster than that of the WT strain on PDA plates either at 26 °C or at 28 °C at all time points from three to nine days post inoculation (Figure 2A). However, the mycelia dry weight of InVir was significantly less than that of WT harvesting from PDB cultured at 26 °C for five days (Figure 2B). 

### 3.3. The InVir Strain Produced More Conidia with a Higher Germination Rate Than the WT Strain

**Figure 3:**
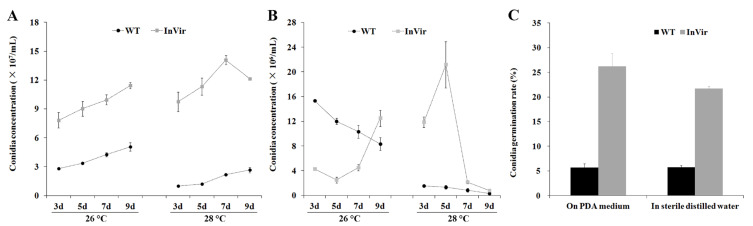
Conidia production and germination of the wildtype strain foc-3b with mild virulence (WT) and its variant Ra-4 with increased virulence (InVir). (**A**,**B**) Cultured in PDB and Armstrong medium, respectively. (**C**) Conidia germination on PDA medium or in sterile distilled water was calculated after 12 h incubation at 26 °C in the dark. Significant differences occurred between WT and InVir for all paired data (*p* < 0.01).

The InVir strain produced significantly more conidia than the WT strain cultured in PDB either at 26 °C or at 28 °C at all time points from three days to nine dpi (Figure 3A). However, conidia production in the Armstrong medium exhibited an intricate process (Figure 3B). The InVir strain produced significantly less conidia than the WT strain in the Armstrong medium at 26 °C from three to seven dpi, while more conidia accumulated nine dpi. At 28 °C, the InVir strain produced significantly more conidia than the WT strain from three to seven dpi and reduced to the same level as the WT strain on nine dpi. Remarkably, the conidia germination rate of InVir was significantly higher than that of WT, incubated at 26 °C in the dark on PDA plates or in sterile distilled water (Figure 3C). 

### 3.4. The InVir Strain Exhibited Variation in Responding to Different Stresses

**Figure 4:**
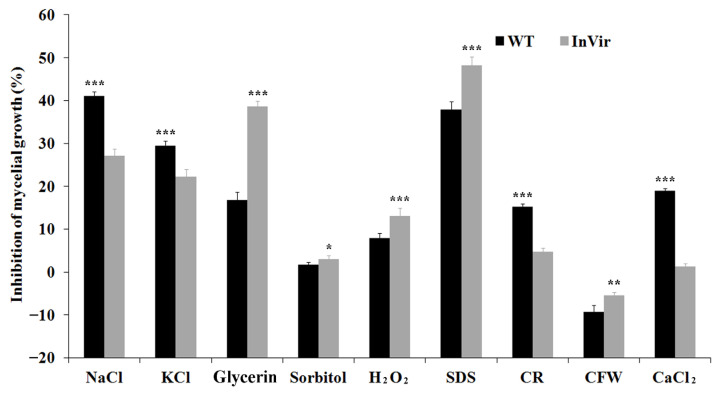
Tolerance to different stresses of the wildtype strain foc-3b with mild virulence (WT) and its variant Ra-4 with increased virulence (InVir). Inhibition of mycelia growth was determined for two strains grown for seven days on PDA medium amended with 1 M NaCl, 1 M KCl, 1 M glycerin, 1 M sorbitol, 0.03% sodium dodecyl sulphate (SDS), 30 mM H_2_O_2_, 0.05% congo red (CR), 0.3 mg/L calcofluor white (CFW), and 0.2 M CaCl_2_, respectively. Significant differences indicated by “*” (*p* < 0.05), “**” (*p* < 0.01) and “***” (*p* < 0.0001).

The mycelial growth of both the WT and the InVir strains of *Foc* was inhibited by various stresses, excluding 0.3 mg/L CFW (Figure 4). Compared to the WT strain, the InVir strain exhibited significantly increased tolerance to osmotic stress caused by 1 M NaCl and 1 M KCl, but decreased tolerance to osmotic stress caused by 1 M glycerin and 1 M sorbitol. The InVir strain exhibited significantly decreased tolerance to oxidative stress generated by 30 mM H_2_O_2_. The InVir strain also showed significantly decreased tolerance to SDS (0.03%) causing cell membrane stress. The InVir strain exhibited inconsistent responses to cell wall stress CR (0.05%) and CFW (0.3 mg/L). The InVir strain also showed significantly increased tolerance to CaCl_2_ (0.2 M).

### 3.5. The InVir Strain Was More Aggressive in Cucumber Than the WT Strain 

**Figure 5:**
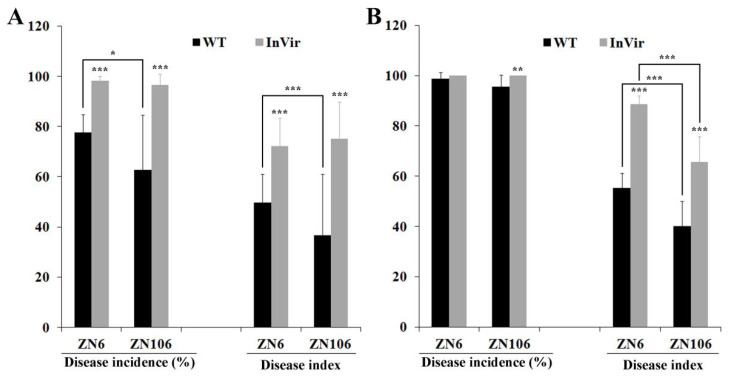
Virulence of the wildtype strain foc-3b with mild virulence (WT) and its variant Ra-4 with increased virulence (InVir). Disease incidence and disease index of susceptible (ZN6) and moderately resistant (ZN106) cucumber cultivars inoculated with WT and InVir strains using seed soaking (**A**) and root-dipping (**B**) methods, respectively. Significant differences indicated by “*” (*p* < 0.05), “**” (*p* < 0.01), and “***” (*p* < 0.0001).

Generally, the InVir strain exhibited higher virulence to both cucumber varieties than the WT strain, regardless of whether inoculation was performed using the seed soaking or root-dipping method (Figure 5). After seed soaking for 14 dpi, the WT strain showed higher virulence to the susceptible cultivar ZN6 than the moderately resistant cultivar ZN106 (Figure 5A). In contrast, the InVir strain showed similar disease incidence and disease severity to the two cultivars. After root dipping for 28 dpi, the two strains did not cause significant differences in disease incidence, but the InVir strain did result in a higher disease severity indicated by disease index compared to the WT strain (Figure 5B). 

### 3.6. The InVir Strain Colonized in the Cucumber Vascular Faster Than the WT Strain

**Figure 6:**
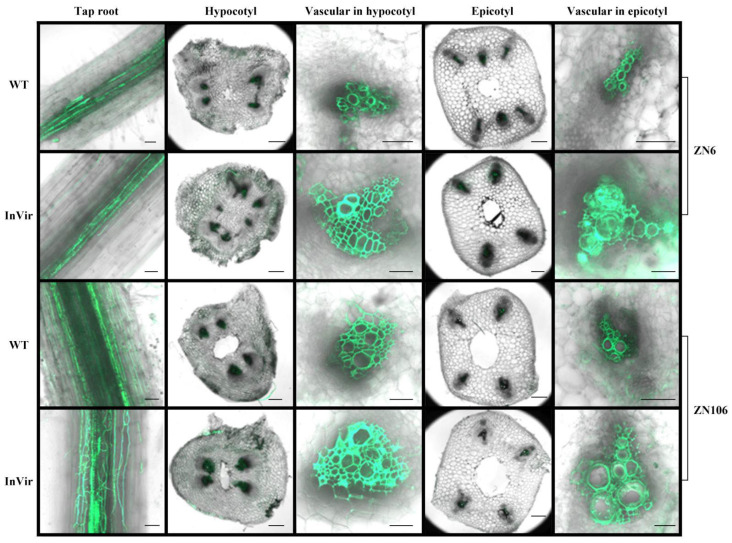
Colonization of the wildtype strain foc-3b with mild virulence (WT) and its variant Ra-4 with increased virulence (InVir) in roots and stems of susceptible (ZN6) and moderately resistant (ZN106) cucumber cultivars. Observations were made seven days after inoculation with wildtype (WT) and InVir strains, which were marked with GFP and showed no changes in virulence. The inoculation process was performed using the root-dipping method. Scale bar = 100 µm (tap root, hypocotyl, and epicotyl) and 20 µm (vascular in hypocotyl and epicotyl).

All cucumber seedlings appeared healthy after seven dpi, but the xylem vessels were colonized by considerable mycelia of both the gInVir and gWT strain (Figure 6). The two strains showed comparable colonization levels in the tap roots of the two cultivars. However, greater accumulation of the fungal mass of the gInVir was observed in vascular tissues of both the hypocotyl and epicotyl than that of the gWT. In particular, the majority of the xylem vessels in the epicotyl of ZN6 cultivar were blocked by the InVir after 14 dpi (Supplementary Figure S1).

## 4. Discussion

Comparing the series of biological characteristics between the wildtype strain foc-3b (WT) and its variant Ra-4 with increased virulence (InVir), we found that improving reproductive efficiency in vitro and in vivo could be a prominent factor for pathogen virulence evolution response to host resistance. This indicates that plant pathogens could use the same strategies to adapt to host pressure, just as the plant or animal hosts usually shorten their life history through earlier maturation and reproduction in response to parasites [23,24].

Inoculum density is critical for successful pathogen infection [25,26]. For a given primary inoculum density, strains with faster and greater sporulation have the advantage of reproducing enough offspring for infection. An investigation of *Zymoseptoria tritici* proved that strains with high reproductive potential are usually more competitive during mixed infection [21]. Not only do the plant pathogens enhance their virulence by improving reproduction efficiency, but also the parasite of the honey bee (*Ascosphaera apis*) has gained enhanced virulence via quicker and earlier spore production [27]. Considering that the fungi persist in producing abundant spores responsible for dispersal and survival during their long evolution history [8], it is understandable how their reproduction efficiency could be improved in such a short time span. 

Conidial germination is a fundamental step in fungal development, in which dormant cells transform into growing hyphae, playing a crucial role in the pathogenesis of host plants [28]. We observed a significant influence of cucumber cultivars on the production and germination of conidia in *F. oxysporum* f. sp. *cucumerium*. Similar results have proven that root exudates from resistant cultivar are more stimulatory for spore germination of *F. oxysporum* f. sp. *pisi* than exudates from susceptible cultivar [29]. In addition to that of *F. oxysporum,* the sporulation capacity of the oat crown rust fungus *Puccinia coronata* f. sp. *avenae* could also be significantly impacted by its host genotypes [30]. In contrast, *F. oxysporum* f. sp. *cubense* spores germinated at a higher rate when exposed to exudates from susceptible banana cultivars compared to resistant cultivars [31], and the same was true of *F. oxysporum* f. sp. *niveum* [32]. Further studies determining the exact stimuli or inhibitors will illustrate the inconsistencies.

The *Foc* strain with enhanced virulence showed inconsistent data in terms of mycelial growth rate on PDA and dry weight in PDB. Based on colony expansion on PDA plates, the InVir strain grew significantly faster than the WT strain. However, in PDB, the InVir strain accumulated less mycelia. It is important to note that conidia were filtered out using three layers of sterilized miracloth before the mycelial dry weight was measured. The reason for the reduced mycelia accumulation in PDB could potentially be attributed to a tradeoff between mycelia growth and conidia production. Consistently, the GFP-marked InVir strain also exhibited increased growth and reproduction in cucumber vascular system (xylem vessels). Although previous research showed *Pyrenophora semeniperda* isolates with faster mycelial growth usually exert less virulence on *Bromus tectorum* seeds [33], the sporulation capacity should also be considered for virulence assessment. 

The growth and reproduction of pathogens within the host play a vital role in assessing their fitness. Apparently, the virulent *Foc* strain propagated more easily in the susceptible cucumber cultivar compared to the resistant cultivar during the later stage after inoculation. In contrast, this phenomenon was not observed for the WT strain, suggesting that resistant hosts may possess stronger defense mechanisms against virulent strains compared to susceptible hosts. A similar trend has been found in Fusarium head blight in the bread wheat pathosystem, in which the fungal mass of the highly aggressive strain MDC_Fg1 of *F. graminearum* was significantly reduced by the resistant cultivars cv. Renan and cv. Cadenza, while the susceptible cultivar Recital did not exhibit the same level of resistance [34]. 

Virulence evolution in fungal pathogens has been extensively studied in the context of genome evolution. In particular, the rapid sequence evolution mediated by transposons has been closely linked to the evolution of fungal plant pathogens [35,36]. The transposons-mediated virulence protein has been identified in fungal wheat pathogens [37]. We also discovered that transposases in the virulence enhanced *Foc* strain were more active than in the WT strain [18]. Whether the active transposons are involved in increasing conidiation in *Foc* has yet to be determined. In future studies, we can delve deeper into the relationship between increased conidiation and genome variation. 

Since resistant cultivars significantly affect pathogen virulence evolution, it is reasonable to grow variable cultivars to slow down the adaptive virulence evolution of pathogens. Using cultivar mixtures for plant disease management has been highly recommended for decades [38,39,40,41], not only due to its benefits in pathogen resistance, but also for yield stability and enhancement [42].

In summary, by comparing the wildtype strain foc-3b (WT) and its virulence-enhanced variant Ra-4 (InVir) in growth, reproduction, stress tolerance, and colonization in cucumber, we determined that increased conidia production and germination in vitro might be associated with the virulence evolution of *Foc*. This study provides valuable insights into the evolution of virulence and enhances our understanding of the underlying mechanisms in the evolutionary biology of *F. oxysporum*.

## Data Availability

All data are included in the main text and Supplementary Materials online.

## Supplementary Materials

The following supporting information can be downloaded at: https://www.mdpi.com/article/10.3390/jof9080847/s1, Figure S1: Observation of colonization by the wildtype strain foc-3b with mild virulence (WT) and its variant Ra-4 with increased virulence (InVir) in roots and stems of susceptible (ZN6) and moderately resistant (ZN106) cucumber cultivars taken 14 days after inoculation.

## References

[1] Gordon T.R., Martyn R.D. (1997). The evolutionary biology of *Fusarium oxysporum*. Annu. Rev. Phytopathol..

[2] Gordon T.R. (2017). *Fusarium oxysporum* and the *Fusarium* wilt syndrome. Annu. Rev. Phytopathol..

[3] Edel-Hermann V., Lecomte C. (2019). Current status of *Fusarium oxysporum* formae speciales and races. Phytopathology.

[4] Zhou X., Wu F. (2012). Dynamics of the diversity of fungal and Fusarium communities during continuous cropping of cucumber in the greenhouse. FEMS Microbiol. Ecol..

[5] Pu Z., Zhang Y., Liu D., Dai L., Wang W. (2011). Research progress in biological control strategies for Fusarium wilt of cucumber. China Veg..

[6] Shen C., Xiong L., Han S., Ma J., Wu J. (2017). The production and circulation of fruit vegetables in China. China Veg..

[7] Shen W., Lin X., Gao N., Zhang H., Yin R., Shi W., Duan Z. (2008). Land use intensification affects soil microbial populations, functional diversity and related suppressiveness of cucumber Fusarium wilt in China’s Yangtze River Delta. Plant Soil.

[8] Stajich J.E., Berbee M.L., Blackwell M., Hibbett D.S., James T.Y., Spatafora J.W., Taylor J.W. (2009). The Fungi. Curr. Biol..

[9] Barriuso J., Hogan D.A., Keshavarz T., Jesus Martinez M. (2018). Role of quorum sensing and chemical communication in fungal biotechnology and pathogenesis. FEMS Microbiol. Rev..

[10] Petriacq P., Stassen J.H.M., Ton J. (2016). Spore density determines infection strategy by the plant pathogenic fungus *Plectosphaerella cucumerina*. Plant Physiol..

[11] Michielse C.B., Rep M. (2009). Pathogen profile update: *Fusarium oxysporum*. Mol. Plant Pathol..

[12] Huang X., Sun M., Lu X., Li S. (2019). Serial passage through resistant and susceptible cucumber cultivars affects the virulence of *Fusarium oxysporum* f. sp. *cucumerinum*. MicrobiologyOpen.

[13] Zhang L., Li S., Miao Z. (2013). Pathogenicity variation and genetic structure differentiation of *Fusarium oxysporum* f. sp. conglutinans in soil under successive cultivation of *Brassica oleracea*. ACTA Phytopathol. Sin..

[14] Jiménez-Gasco M.M., Navas-Cortés J.A., Jiménez-Díaz R.M. (2004). The *Fusarium oxysporum* f. sp. *ciceris/Cicer arietinum* pathosystem: A case study of the evolution of plant-pathogenic fungi into races and pathotypes. Int. Microbiol..

[15] Wang B., Brubaker C.L., Tate W., Woods M.J., Burdon J.J. (2008). Evolution of virulence in *Fusarium oxysporum* f. sp. *vasinfectum* using serial passage assays through susceptible cotton. Phytopathology.

[16] Harris J.M., Balint-Kurti P., Bede J.C., Day B., Gold S., Goss E.M., Grenville-Briggs L.J., Jones K.M., Wang A., Wang Y. (2020). What are the top 10 unanswered questions in molecular plant-microbe interactions?. Mol. Plant-Microbe Interact..

[17] Sacristán S., Goss E.M., Eves-van den Akker S. (2021). How do pathogens evolve novel virulence activities?. Mol. Plant-Microbe Interact..

[18] Huang X.Q., Lu X.H., Sun M.H., Guo R.J., Van Diepeningen A.D., Li S.D. (2019). Transcriptome analysis of virulence-differentiated *Fusarium oxysporum* f. sp. *cucumerinum* isolates during cucumber colonisation reveals pathogenicity profiles. BMC Genom..

[19] Barrett L.G., Bell T., Dwyer G., Bergelson J. (2011). Cheating, trade-offs and the evolution of aggressiveness in a natural pathogen population. Ecol Lett..

[20] Paisely D., Robson G.D., Denning D.W. (2005). Correlation between in vitro growth rate and in vivo virulence in *Aspergillus fumigatus*. Medical Mycol..

[21] Pagán I., Alonso-Blanco C., García-Arenal F. (2008). Host responses in life-history traits and tolerance to virus infection in *Arabidopsis thaliana*. PLoS Pathog..

[22] Vakalounakis D.J., Wang Z., Fragkiadakis G.A., Skaracis G.N., Li D.-B. (2004). Characterization of *Fusarium oxysporum* isolates obtained from cucumber in China by pathogenicity, VCG, and RAPD. Plant Dis..

[23] Bernasconi A., Alassimone J., McDonald B.A., Sanchez-Vallet A. (2022). Asexual reproductive potential trumps virulence as a predictor of competitive ability in mixed infections. Environ. Microbiol..

[24] Ohlberger J., Langangen O., Edeline E., Olsen E.M., Winfield I.J., Fletcher J.M., Ben James J., Stenseth N.C., Vollestad L.A. (2011). Pathogen-induced rapid evolution in a vertebrate life-history trait. Proc. R. Soc. B-Biol. Sci..

[25] DeVay J.E., Gutierrez A.P., Pullman G.S., Wakeman R.J., Garber R.H., Jeffers D.P., Smith S.N., Goodell P.B., Roberts P.A. (1997). Inoculum densities of *Fusarium oxysporum* f. sp. *vasinfectum* and *Meloidogyne incognita* in relation to the development of Fusarium wilt and the phenology of cotton plants (*Gossypium hirsutum*). Phytopathology.

[26] Tooley P.W., Browning M., Leighty R.M. (2013). Inoculum density relationships for infection of some Eastern US forest species by *Phytophthora ramorum*. J. Phytopathol..

[27] Evison S.E.F., Foley K., Jensen A.B., Hughes W.O.H. (2015). Genetic diversity, virulence and fitness evolution in an obligate fungal parasite of bees. J. Evol. Biol..

[28] Baltussen T.J.H., Zoll J., Verweij P.E., Melchers W.J.G. (2020). Molecular mechanisms of conidial germination in *Aspergillus* spp.. Microbiol. Mol. Biol. Rev..

[29] Buxton E.W. (1957). Some effects of pea root exudates on physiologic races of *Fusarium oxysporum* Fr. f. *pisi* (Linf.). Trans. Br. Mycol. Soc..

[30] Bruns E., Carson M., May G. (2012). Pathogen and host genotype differently affect pathogen fitness through their effects on different life-history stages. BMC Evol. Biol..

[31] Li C., Chen S., Zuo C., Sun Q., Ye Q., Yi G., Huang B. (2011). The use of GFP-transformed isolates to study infection of banana with *Fusarium oxysporum* f. sp. *cubense* race 4. Eur. J. Plant Pathol..

[32] Wu F., Liu B., Zhou X. (2010). Effects of root exudates of watermelon cultivars differing in resistance to Fusarium wilt on the growth and development of *Fusarium oxysporum* f. sp. *niveum*. Allelopathy J..

[33] Meyer S.E., Stewart T.E., Clement S. (2010). The quick and the deadly: Growth vs virulence in a seed bank pathogen. New Phytol..

[34] Fabre F., Bormann J., Urbach S., Roche S., Langin T., Bonhomme L. (2019). Unbalanced roles of fungal aggressiveness and host cultivars in the establishment of the Fusarium head blight in bread wheat. Front. Microbiol..

[35] Faino L., Seidl M.F., Shi-Kunne X., Pauper M., Van Den Berg G.C.M., Wittenberg A.H.J., Thomma B.P.H.J. (2016). Transposons passively and actively contribute to evolution of the two-speed genome of a fungal pathogen. Genome Res..

[36] Singh N.K., Badet T., Abraham L., Croll D. (2021). Rapid sequence evolution driven by transposable elements at a virulence locus in a fungal wheat pathogen. BMC Genom..

[37] McDonald M.C., Taranto A.P., Hill E., Schwessinger B., Liu Z., Simpfendorfer S., Milgate A., Solomon P.S. (2019). Transposon-mediated horizontal transfer of the host-specific virulence protein ToxA between three fungal wheat pathogens. mBio.

[38] Mundt C.C. (2002). Use of multiline cultivars and cultivar mixtures for disease management. Annu. Rev. Phytopathol..

[39] Clin P., Grognard F., Andrivon D., Mailleret L., Hamelin F.M. (2022). Host mixtures for plant disease control: Benefits from pathogen selection and immune priming. Evol. Appl..

[40] Mikaberidze A., McDonald B.A., Bonhoeffer S. (2015). Developing smarter host mixtures to control plant disease. Plant Pathol..

[41] Garrett K.A., Mundt C.C. (2000). Host diversity can reduce potato late blight severity for focal and general patterns of primary inoculum. Phytopathology.

[42] Wuest S.E., Peter R., Niklaus P.A. (2021). Ecological and evolutionary approaches to improving crop variety mixtures. Nat. Ecol. Evol..

