# Supplementary figures and images for: Increased Conidia Production and Germination In Vitro Correlate with Virulence Enhancement in Fusarium oxysporum f. sp. cucumerinum
Source: J Fungi (Basel). 2023 Aug 14;9(8):847. doi: 10.3390/jof9080847 (PMC10455488; doi:10.3390/jof9080847)

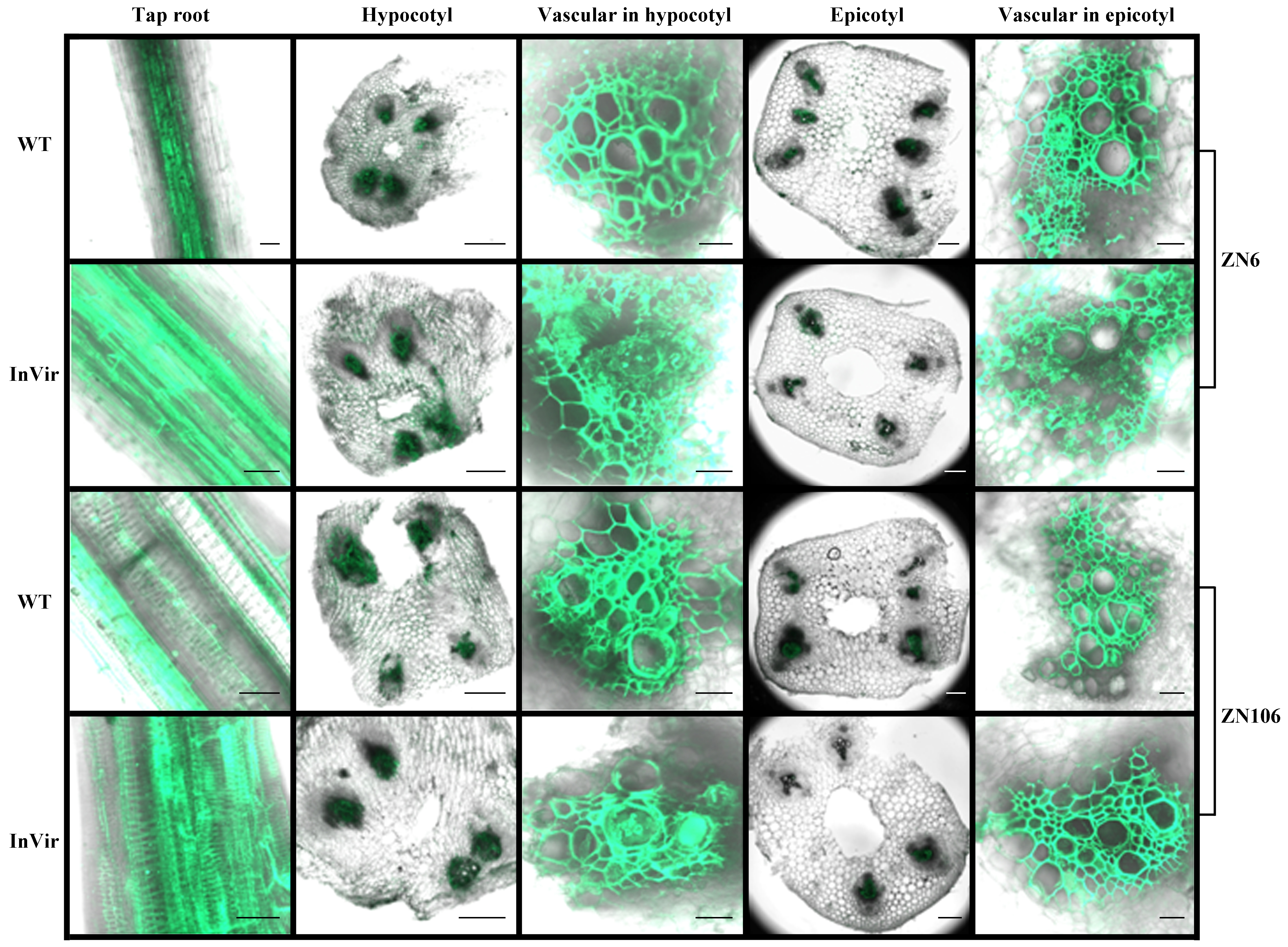

Supplement: Supplementary file 1 [file jof-09-00847-s001.zip › Figure S1.jpg]
